# Supplementary figures and images for: Energetic substrate availability regulates synchronous activity in an excitatory neural network
Source: PLoS One. 2019 Aug 13;14(8):e0220937. doi: 10.1371/journal.pone.0220937 (PMC6692003; doi:10.1371/journal.pone.0220937)

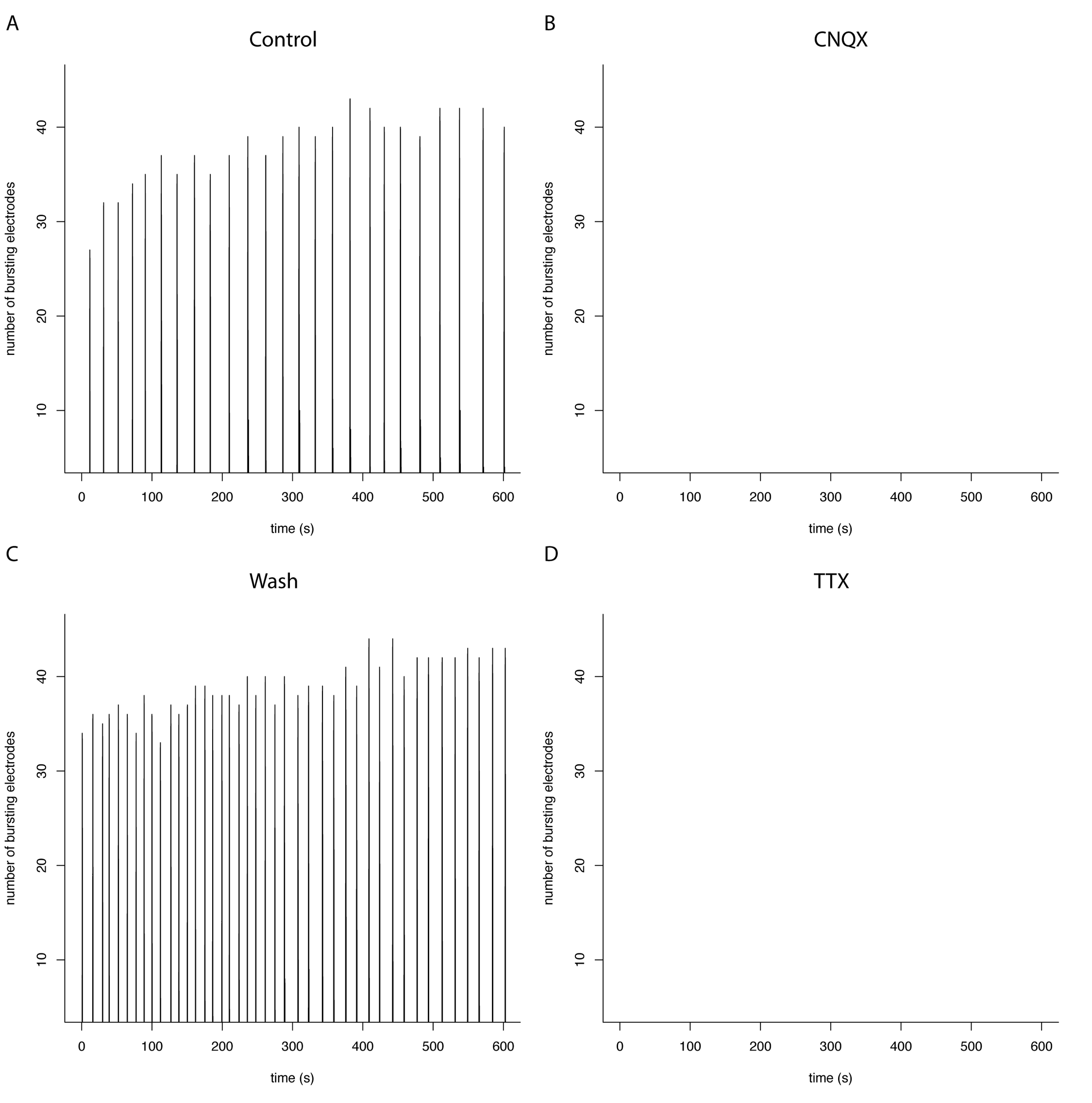

Supplement: S1 Fig — Representational examples of the recorded data used to generate Fig 1A in main text. These are intended to complement Supplementary Dataset 1 in Figshare (each panel has an associated 600sec MEA recording). The plots represent the number of electrodes bursting across the MEA at the given time point under the given experimental conditions: A) control; B) CNQX; C) wash; and D) TTX. (TIF) [file pone.0220937.s001.tif]

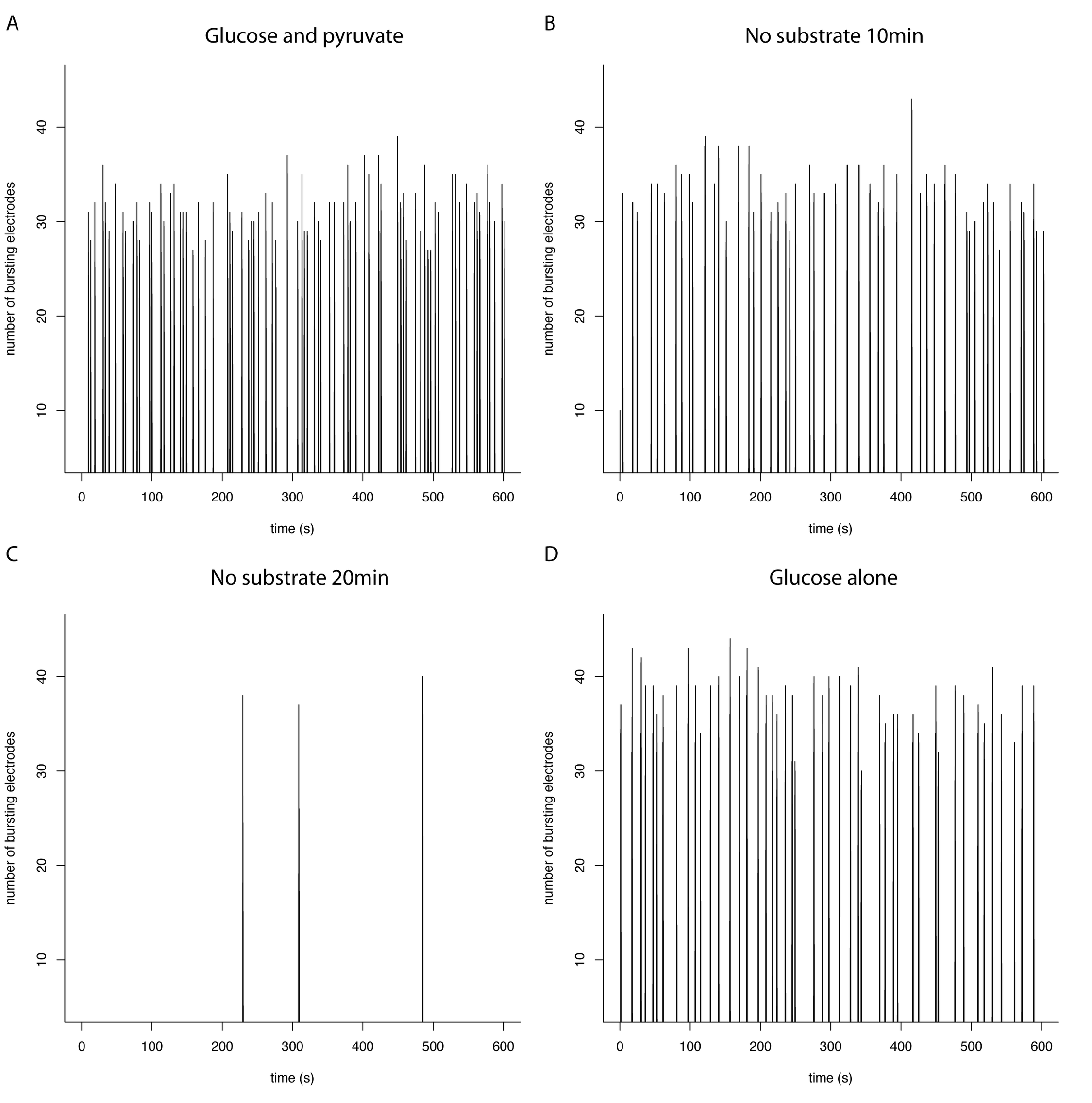

Supplement: S2 Fig — Representational examples of the recorded data used to generate Fig 3B in main text. These are intended to complement Supplementary Dataset 2 in Figshare (each panel has an associated 600sec MEA recording). The plots represent the number of electrodes bursting across the MEA at the given time point under the given experimental conditions: A) glucose and pyruvate; B) no substrate 10min; C) no substrate 20min; and D) glucose alone. (TIF) [file pone.0220937.s002.tif]

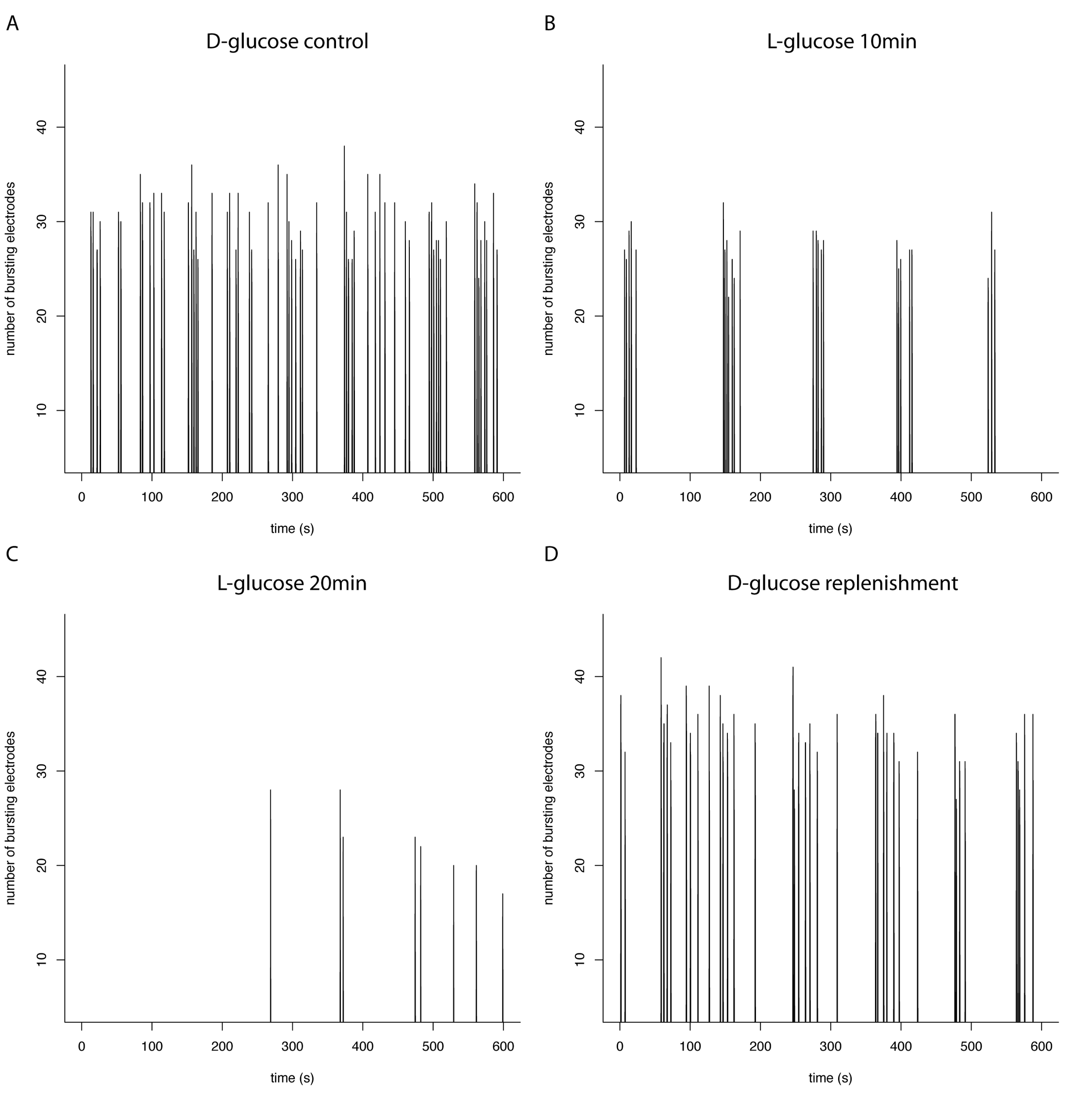

Supplement: S3 Fig — Representational examples of the recorded data used to generate Fig 3C in main text. These are intended to complement Supplementary Dataset 3 in Figshare (each panel has an associated 600sec MEA recording). The plots represent the number of electrodes bursting across the MEA at the given time point under the given experimental conditions: A) D-glucose control; B) L-glucose 10min; C) L-glucose 20min; and D) D-glucose replenishment. (TIF) [file pone.0220937.s003.tif]

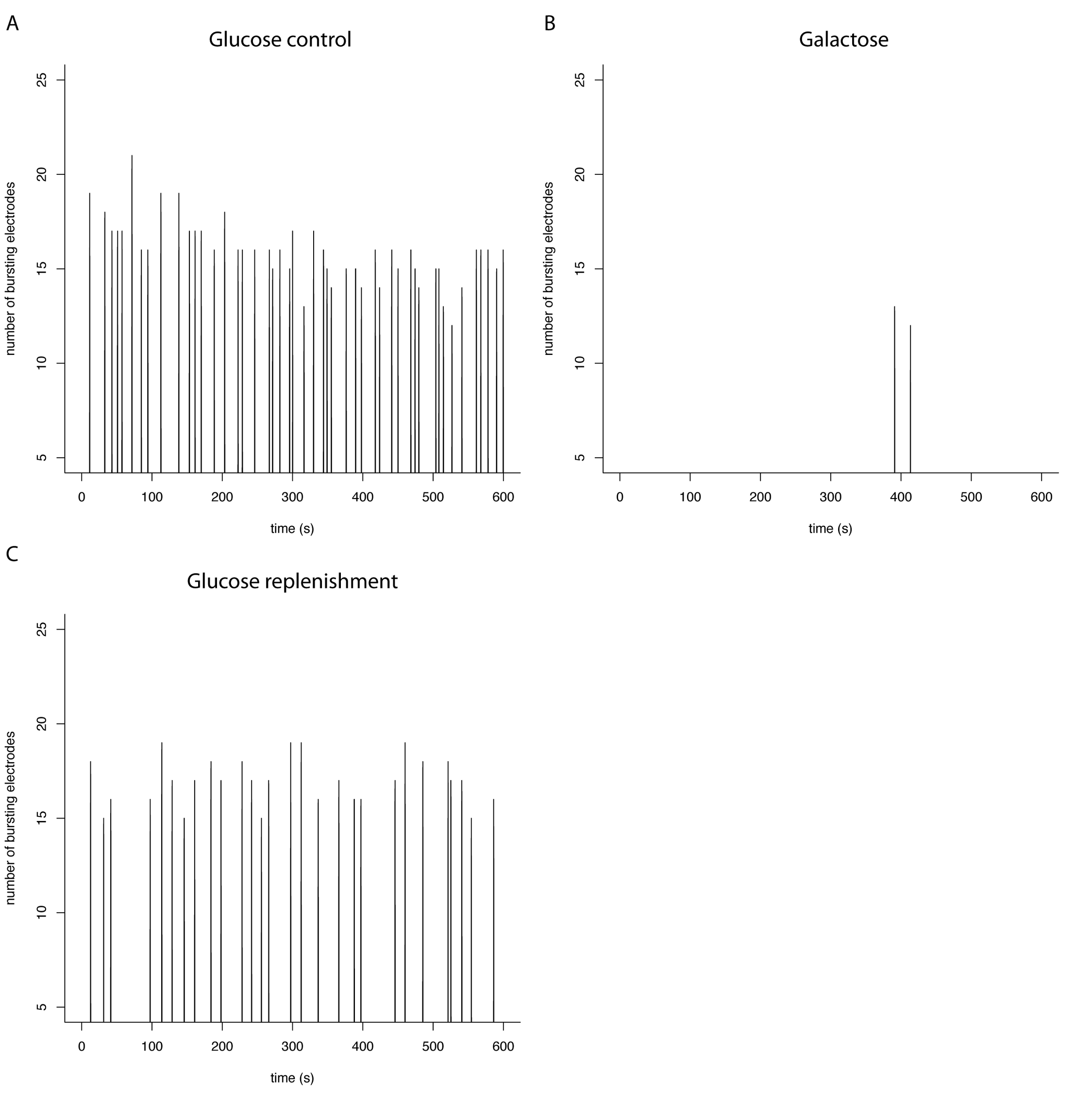

Supplement: S4 Fig — Representational examples of recorded data used to study the effects of replacing 5mM glucose with 5mM galactose on synchronous bursting. These are intended to complement Supplementary Dataset 4 in Figshare (each panel has an associated 600sec MEA recording). The plots represent the number of electrodes bursting across the MEA at the given time point under the given experimental conditions: A) glucose control; B) galactose; and C) glucose replenishment. (TIF) [file pone.0220937.s004.tif]
